# MCPmed: a call for Model Context Protocol-enabled bioinformatics web services for LLM-driven discovery

**DOI:** 10.1093/bib/bbag076

**Published:** 2026-02-23

**Authors:** Matthias Flotho, Ian Ferenc Diks, Philipp Flotho, Leidy-Alejandra G Molano, Pascal Hirsch, Andreas Keller

**Affiliations:** Chair for Clinical Bioinformatics, Center for Bioinformatics, Saarland University, Saarland University Campus, 66123 Saarland, Germany; Helmholtz Institute for Pharmaceutical Research Saarland (HIPS), Saarland University, Saarland University Campus, 66123 Saarland, Germany; Chair for Clinical Bioinformatics, Center for Bioinformatics, Saarland University, Saarland University Campus, 66123 Saarland, Germany; Helmholtz Institute for Pharmaceutical Research Saarland (HIPS), Saarland University, Saarland University Campus, 66123 Saarland, Germany; Chair for Clinical Bioinformatics, Center for Bioinformatics, Saarland University, Saarland University Campus, 66123 Saarland, Germany; Chair for Clinical Bioinformatics, Center for Bioinformatics, Saarland University, Saarland University Campus, 66123 Saarland, Germany; Chair for Clinical Bioinformatics, Center for Bioinformatics, Saarland University, Saarland University Campus, 66123 Saarland, Germany; Chair for Clinical Bioinformatics, Center for Bioinformatics, Saarland University, Saarland University Campus, 66123 Saarland, Germany; Helmholtz Institute for Pharmaceutical Research Saarland (HIPS), Saarland University, Saarland University Campus, 66123 Saarland, Germany; Pharma Science Hub (PSH), Saarland University Campus, Saarland University Campus, 66123 Saarland, Germany

**Keywords:** MCP, database, web server, GEO, API, LLM

## Abstract

Bioinformatics web servers are critical resources in modern biomedical research, facilitating interactive exploration of datasets through custom-built interfaces with rich visualization capabilities. However, this mostly human-centric design limits machine readability for large language models (LLMs) and deep research agents. We address this gap by adapting model context protocol (MCP) to bioinformatics web server backends, a standardized, machine-actionable layer that explicitly associates web service endpoints with scientific concepts and detailed metadata. Our implementations across widely used databases (GEO, STRING, and UCSC Cell Browser) demonstrate enhanced exploration capabilities through MCP-enabled LLMs. To accelerate adoption, we propose MCPmed, a community effort supplemented by lightweight *breadcrumbs* for services not yet fully MCP-enabled and templates for setting up new servers. This structured transition aims to significantly enhance automation, reproducibility, and interoperability, preparing bioinformatics web services for next-generation research agents.

## Graphical Abstract

**Graphical Abstract:**
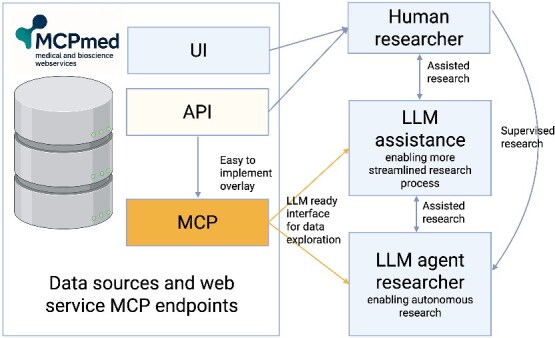

## Introduction

Traditional bioinformatics web servers have been designed primarily for human users and have long been evaluated according to nucleic acids research (NAR) guidelines that emphasize uptime and citation-friendly uniform resource locators (URLs). However, the growing use of autonomous research agents built on large language models (LLMs) highlights an additional critical requirement: bioinformatics services should be designed to be both human- and machine-actionable, in line with the FAIR principles of findability, accessibility, interoperability, and reusability [1]. While traditional application programming interface (APIs) have enabled partial automation, they remain fragmented and inconsistently documented, making seamless cross-service integration difficult. This motivates the development of a more standardized interoperability layer such as the model context protocol (MCP), which allows LLMs to reliably query, combine, and interpret bioinformatics services without brittle workarounds.

Recent advancements in LLMs and autonomous research agents underscore the urgent need for bioinformatics web servers to evolve from predominantly human-oriented interfaces toward fully machine-actionable platforms. Recent efforts show promising progress in automated research and data exploration [2–4]. The MCP [5] directly addresses this challenge by providing a standardized semantic contract layered over existing application programming interface (API) specifications. MCP explicitly associates each API endpoint with scientific concepts, along with versioned metadata, facilitating automated discovery, invocation, and verification of web services. In particular, several practical benefits arise instantly from adapting to MCP. In practical terms, MCP represents each operation as a “tool” with a JSON Schema specification for its inputs and outputs. These tools can be annotated with concept identifiers drawn from general vocabularies such as Schema.org and from domain ontologies including Bioschemas [6], EDAM [7], Gene Ontology (GO) [8], or other OBO [9] resources. MCP itself remains vocabulary-agnostic: it merely provides structured attachment points for such identifiers, enabling communities to choose and evolve appropriate domain-specific semantics. Those include enhanced **automation**, enabling improved integration between data discovery, computational analysis, and workflow documentation, improved **reproducibility**, achieved through concept-level versioning and audit-ready parameter tracking, and increased **interoperability**, as shared MCP concepts become standardized across institutions, analogous to Global Alliance for Genomics and Health (GA4GH) tool registry service (TRS) in genomics [10] (Table 1).

**Table 1:** Comparative summary: FAIR vs. GA4GH TRS vs. MCP

	FAIR principles	GA4GH TRS	MCP
**Goal**	Improve data reuse by making data findable, accessible, interoperable, and reusable	Standard for listing, describing, and discovering genomics tools and workflows across registries	Standardize secure connections between AI applications and external data sources/tools
**Scope**	Conceptual framework and principles for data management	Specific API schema for genomics tool/workflow registration and discovery	Open protocol for connecting LLMs to external resources
**Machine actionability**	Encouraged but not prescriptive about implementations	Well-defined API schemas with structured endpoints	High priority: designed for parsing by LLMs and AI agents
**Technical representation**	Abstract principles for describing data and metadata, not a specific encoding	Structured JSON schemas describing tools and workflows and their associated metadata	JSON-based protocol specifications describing tools, resources, and operations (protocol-level metadata, complementary to data-level metadata)
**Governance**	Broad community acceptance, decentralized approach	Managed by GA4GH organization with established processes	Open standard introduced by Anthropic (Nov 2024), gaining adoption
**Domain focus**	General data management across disciplines	Genomics and bioinformatics tools	AI/LLM integration with external systems
**Examples**	GEO persistent identifiers (GSE12345), DOI linking, standardized metadata formats	Dockstore, WorkflowHub registries; CWL/WDL workflow descriptors	GEOmcp, STRING MCP, UCSC Cell Browser MCP enabling LLM-driven queries and cross-service integration
**Relationship to MCP**	MCP could operationalize FAIR principles for AI systems	MCP could enhance TRS outputs for autonomous AI agents	Complements existing frameworks by providing AI-specific integration layers

In this manuscript, we illustrate MCP’s transformative potential through practical implementations. First, we introduce MCPmed, including diverse MCP layers for highly used databases, such as a lightweight MCP layer for the Gene Expression Omnibus (GEO) [11], enabling LLMs to autonomously search and retrieve data via existing GEO API endpoints. Second, we propose a simple hypertext markup language (HTML) metadata system *breadcrumbs* to bridge existing services towards MCP readiness. These examples demonstrate how MCP adoption can rapidly transition bioinformatics web servers into integral, fully automated components of next-generation biomedical research workflows.

**Figure 1:**
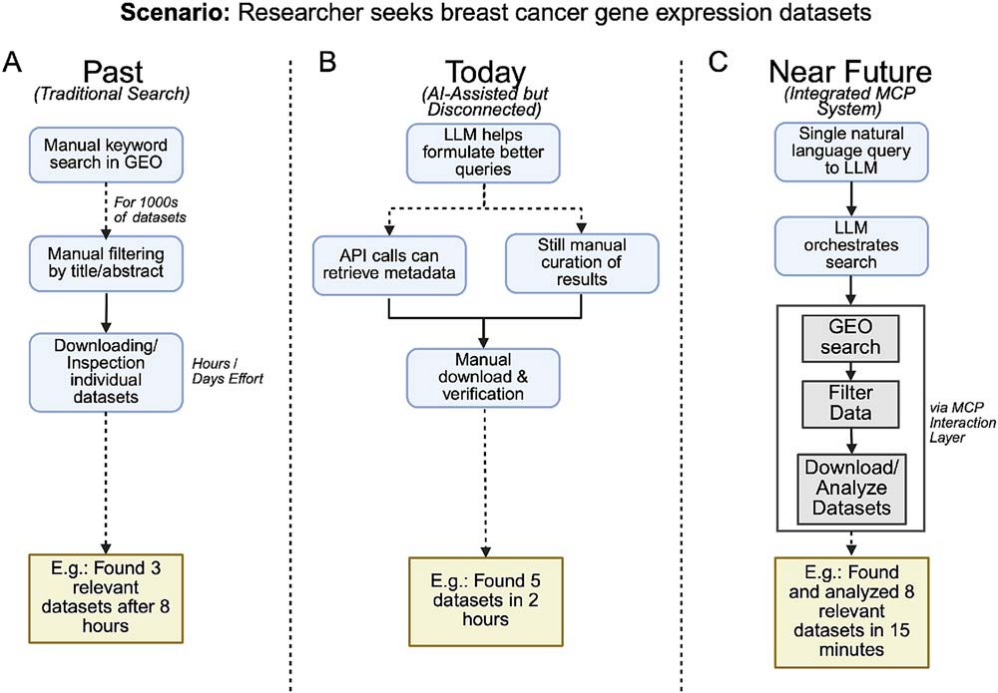
Evolution of hypothesis-driven bioinformatics workflows in a breast cancer gene expression dataset discovery case study, comparing (A) traditional manual GEO searches requiring extensive manual filtering and dataset inspection, (B) current AI-assisted but disconnected workflows with LLM-supported querying and partial automation yet continued manual integration, and (C) a near-future integrated MCP system enabling end-to-end automated search, filtering, download, and initial analysis through a single natural language query. Figures created with Biorender.com.

By adopting MCP, biomedical web services can effectively address the emerging requirements of enhanced research workflows, supported by systems capable of improved data discovery and analysis integration (Fig. 1).

## Today’s bioinformatics is FAIR

The publication of the FAIR Guiding Principles in 2016 [1] marked a major milestone in scientific data stewardship, aiming to make digital assets FAIR. These principles emphasize machine-actionability, i.e. enabling computational systems to find, access, and reuse data with minimal human intervention, because of the increasing scale and complexity of research data. However, FAIR primarily ensures that data and metadata can be retrieved and processed by computers; it does not require that a computer can autonomously determine how a dataset relates to scientific concepts. For example, FAIR-compliant metadata may support indexing and identifier resolution, but there is no obligation that services expose stable, semantically linked APIs that allow automated agents to interpret content in a domain-aware way.

This distinction is visible in practice: community guidelines such as those of the NAR Web Server and Database Issues [12, 13] recognize human-centric portals as FAIR-compliant even when no consistent, machine-oriented API is available. As a result, automated workflows often rely on brittle strategies such as scraping HTML or adapting to ad hoc JSON endpoints, which hinders scalability and reproducibility. While FAIR and GA4GH TRS have greatly advanced accessibility and interoperability, neither addresses the need for AI-ready semantics and standardized machine-facing interfaces across use cases.

We therefore see MCP not as a replacement for FAIR, but as a complementary integration layer designed to enable robust, LLM-driven automation. Table 1 contrasts FAIR, GA4GH TRS, and MCP, highlighting MCPmed’s unique contribution: broad applicability, explicit AI integration, and configurable metadata hooks that support provenance, runtime adaptation, and monitoring without sacrificing interoperability. In this sense, adopting MCP closes a crucial gap, transforming FAIR-compliant but human-centered resources into machine-actionable services that autonomous research agents can discover, interpret, and use reliably.

## Challenges and opportunities for model context protocol-native bioinformatics

Today’s bioinformatics landscape offers powerful capabilities but remains fragmented. This fragmentation is characterized by diverse API styles (REST+JSON, GraphQL, SOAP, and HTML forms), varied authentication protocols, and inconsistent pagination. Highly specialized services, such as harmonized data collections (UCSC Cell Browser [14], ZEBRA [15], DISCO [16], and miRNATissueAtlas [17]), general databases without harmonization, such as GEO [11], genome alignment engines (M1CR0B1AL1Z3R [18]), data extraction platforms (miRMASTER [19]), and sophisticated visualization tools (miRTargetLink [20]) are challenging for autonomous agents to utilize without customized integration. By enforcing API specifications combined with semantic MCP alignment, we provide autonomous agents a unified grammar for service discovery, invocation, and error handling. Concretely, each MCP tool is described by a JSON Schema for its input and output parameters and is associated with one or more high-level scientific concept tags (e.g. gene_expression_series or microglia_depletion_dataset). These concept tags are designed to carry ontology-backed identifiers from resources such as Schema.org, Bioschemas, EDAM, GO, or other OBO ontologies, so that autonomous agents can reason not only about how to call an endpoint, but also about what biological object or operation it represents. Standardized manifests surface details such as rate limits, authentication, and pagination clearly, eliminating ad-hoc code and fragile scraping routines [21]. Operational reliability further enhances this model: services can implement explicit health checks and service-level commitments within their configurations, enabling autonomous pipelines to manage downtime proactively, queue tasks efficiently, or dynamically shift to alternative providers. MCP requires servers to implement proper capability negotiation and error handling mechanisms to ensure reliable communication between hosts and servers.

### From human-centric portals to model context protocol-native infrastructures

The rise of LLMs necessitates web services optimized for autonomous agents. To satisfy both human and machine users, bioinformatics servers should implement three sequential layers:

- **User interface (UI)**: browser-based, intuitive interfaces for exploratory tasks and clear documentation.
- **API layer**: standardized, machine-readable descriptions defining exact usage, parameters, and error handling.
- **MCP layer**: semantic metadata tagging each API endpoint with scientific concepts and providing explicit model/version provenance.

This layered approach (UI $\rightarrow $ API $\rightarrow $ MCP) ensures human accessibility while enabling autonomous execution and complete reproducibility, addressing the requirements of next-generation bioinformatics research infrastructures.

**Figure 2:**
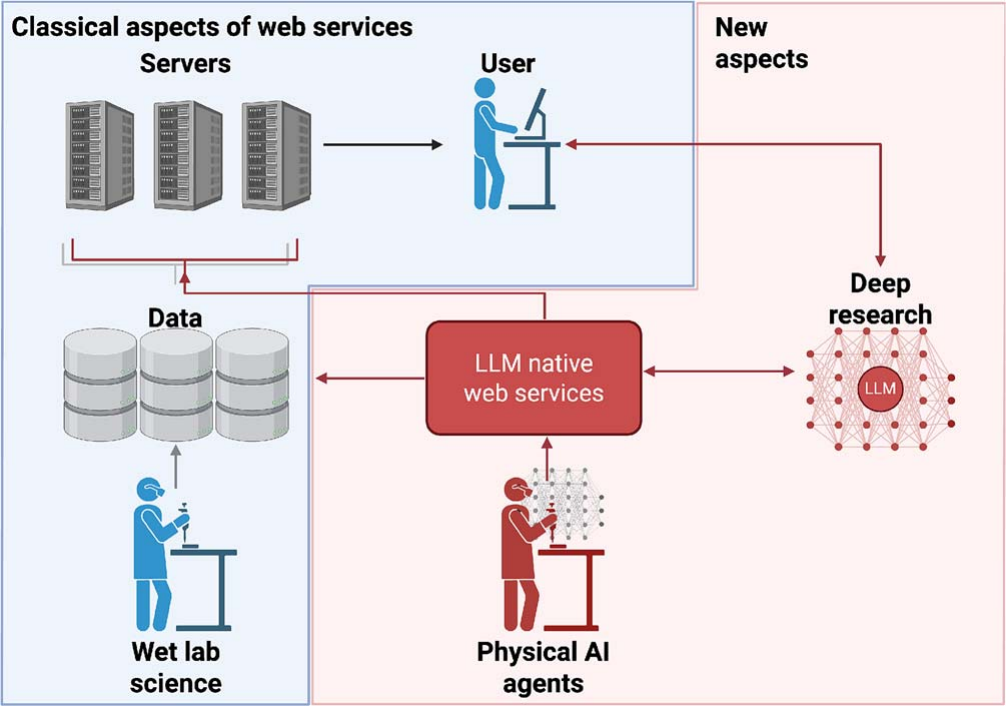
Evolution of a bioinformatics server as it acquires agent-readiness. The left panel shows a classic UI-only service. In the center, an API layer makes the same service callable by a script or language model. The rightmost panel adds an endpoint to a plug-and-play module in fully automated wet- plus dry-lab pipelines.

Figure 2 illustrates the complementary facets we foresee for the next generation of bioinformatics web services paving the road towards fully automated research pipelines.

## Enhancing data reuse through model context protocol-enabled discovery

An important and well-recognized challenge in current bioinformatics practice is that publications necessarily emphasize a subset of analyses and results to communicate a clear narrative, even when the underlying datasets support many additional downstream questions. As a practical consequence, subsequent discovery and reuse are often guided by unstructured metadata and a small number of prominently described signals, rather than by systematic access to the full data corpus. Already in 2013 Piwowar and Vision [22] described that most of the 1.3 M human omics samples deposited on GEO remain *acutely underused* because discovery often relies on unstructured metadata [22, 23]. This under-exploitation occurs even for well-curated datasets with accompanying web interfaces. For example, Hahn *et al*. [24] provide a rich resource to study aging trajectories in mice, but—as is typical—only a subset of possible questions is pursued and emphasized in the primary publication. In practice, many additional analyses (e.g. alternative cell-type-, region-, or pathway-focused queries; cross-study comparisons; or orthogonal validation questions) remain latent because they require repeated manual search, download, and reformatting steps across resources.

We propose an evolutionary enhancement in data usability that aligns bioinformatics web services with current LLM capabilities: very large bioinformatics datasets contain potentially far more latent insights than can be exploited using current approaches, and MCP can massively improve discovery and retrieval efficiency—thereby enabling extraction of more of these latent findings. Rather than changing how scientists choose focused questions, MCP lowers the operational cost of exploring additional, well-motivated hypotheses against existing data corpora. With minimal effort, essentially any web service or database with an existing API can be included in the LLM-assisted research process using MCPs. To demonstrate this point, we implemented an easy-to-use MCP client layering the GEO database enabling automated search for datasets supporting hypotheses, but also download the data. In combination with MCPs currently developed such as the single-cell MCP [25], this might already be the base for a fully automated workflow for backing up findings with autonomous data retrieval and analysis.

## Comparison with existing bioinformatics integration platforms

Several platforms have previously attempted to standardize bioinformatics tool integration, each addressing different aspects of the interoperability challenge. The **GA4GH TRS** [10] provides a standardized API for discovering and describing genomics tools and workflows, enabling cross-platform tool sharing. However, TRS focuses primarily on workflow registration and metadata rather than real-time LLM interaction and semantic concept mapping that MCP provides.

Web-based platforms such as **GREIN** [26] have enhanced GEO data reusability through interactive re-analysis interfaces, while systematic metadata annotation efforts [23] have improved dataset discoverability. These approaches successfully address human-centered data exploration but lack the standardized machine-actionable interfaces required for autonomous agent integration.

The **GA4GH ecosystem** more broadly, including standards like Beacon and Data Repository Service (DRS), has established important precedents for federated genomic data access. MCP complements these efforts by providing an LLM-native integration layer that can expose GA4GH-compliant services to autonomous research agents without replacing existing standards.

What distinguishes MCP from these platforms is its explicit design for **LLM integration**: semantic concept mapping, explicit versioning metadata, and standardized error handling optimized for agent-based reasoning. While TRS enables *tool discovery*, MCP enables *autonomous tool invocation and chaining*. Where GREIN provides *interactive interfaces*, MCP provides *programmatic semantic contracts*. MCP thus occupies a complementary niche as an AI-specific integration layer that can operate alongside existing bioinformatics standards.

Our GEOmcp and STRING DB implementations demonstrate this complementarity: they layer MCP semantics over existing APIs (NCBI E-utilities, STRING REST API) without requiring fundamental infrastructure changes. This approach allows gradual adoption while preserving investments in existing platforms.

## Methods

### GEOmcp

GEOmcp was developed as a proof-of-concept MCP implementation to demonstrate the practical utility of machine-actionable bioinformatics services. The system provides a standardized interface between LLMs and the NCBI GEO through three integrated components: NCBI E-utilities API integration, MCP protocol implementation, and optimized LLM interaction layers.

#### NCBI E-utilities integration and rate limiting

The core data access layer utilizes NCBI’s E-utilities API with mandatory email registration and optional API key authentication for enhanced rate limits. Two primary endpoints were integrated: ESearch for querying GEO databases (gds, geoprofiles) and Esummary for retrieving detailed record metadata. Rate limiting implements 0.1 s delays between requests to ensure NCBI compliance with automatic API key detection enabling up to 10 requests per second when available.

Authentication parameters are managed through JSON configuration files located at ~/.geo-mcp/config.json, following established bioinformatics tool conventions for credentials management.

#### Model context protocol schema design and tool definitions

Eight distinct tools were implemented, each with JSON Schema specification defining input parameters, output structure, and semantic descriptions optimized for LLM interpretation:

**Core search functions:** Universal search across all GEO record types (search_geo), targeted searches for gene expression profiles (search_geo_profiles), curated datasets (search_geo_datasets), experimental series (search_geo_series), individual samples (search_geo_samples), and platform definitions (search_geo_platforms).

**Data management functions:** Automated Simple Omnibus Format in Text (SOFT) file [27] downloads (download_geo_data) and download status monitoring (get_download_status).

Tool schemas follow MCP specifications with required and optional parameters clearly defined. The universal search function implements automatic result categorization by GEO accession profiles (GSE, GSM, GPL, and GDS), enabling LLMs to understand data structure and formulate contextually appropriate follow-up queries.

#### Large language model interface optimization

The interface design prioritizes semantic clarity over technical complexity, allowing LLMs to generate biologically meaningful queries without detailed knowledge of GEO database architecture. Each tool returns structured JSON responses wrapped in MCP TextContext objects, supporting three key LLM capabilities: autonomous query refinement through iterative search term adjustment, contextual parameter selection for result limits and filtering, and cross-reference integration linking related GEO record types.

Response formatting emphasizes human-readable descriptions alongside machine-actionable identifiers, facilitating both automated processing and user verification of LLM-generated queries.

#### Download management and safety controls

The download subsystem implements comprehensive safety measures including path validation restricting operations to predefined directories, configurable size limits (5 GB per file, 10 GB total storage), semaphore-controlled concurrent downloads (default: three parallel transfers), and automatic XML metadata preservation alongside SOFT archives.

Asynchronous HTTP requests with configurable timeouts (300 s default) include disk space verification before transfer initiation, ensuring system stability during large dataset acquisitions.

#### Error handling and system resilience

Comprehensive error handling addresses API failures through graceful degradation with informative messages, automatic NCBI rate limit compliance, configuration fallbacks with user warnings, and configurable network timeout management. This design ensures continued functionality despite common failure modes in distributed bioinformatics infrastructure.

#### Deployment architecture

Two deployment modes support different integration scenarios: MCP stdio mode for direct integration with Claude Desktop and compatible MCP clients, and HTTP server mode providing REST API endpoints for web-based integrations and testing environments. The HTTP implementation includes CORS support, OpenAPI documentation generation, and Server-Sent Events for real-time operation monitoring.

#### Performance evaluation and benchmarking

Initial performance evaluation demonstrates processing capabilities of 2–5 s for typical LLM queries returning 10–50 results, with performance primarily constrained by NCBI E-utilities response latency rather than local processing overhead. The MCP layer contributes minimal additional latency (<100 ms) compared with direct API calls, validating the efficiency of the protocol implementation.

#### Implementation availability

The complete GEOmcp implementation is distributed as an open-source Python package (geo-mcp) via PyPI, supporting Python 3.10+ environments. Deployment requires single-command installation followed by interactive configuration setup, enabling adoption, and testing by the bioinformatics community.

### Breadcrumbs implementation

#### Breadcrumbs discovery system

To enable LLM interaction with services lacking native MCP support, we developed a lightweight discovery mechanism called “breadcrumbs”, structured metadata embedded as HTML comments within service web pages.

#### Implementation architecture

The breadcrumbs system consists of three components: (i) HTML-embedded JSON metadata following a standardized schema, (ii) an MCP server implementing the discovery protocol, and (iii) client-side tools enabling direct API interaction.

The MCP server was implemented using the MCP SDK (v1.18.2) with TypeScript, utilizing Zod (TypeScript schema validation library) schemas [28] for runtime validation. The server exposes three primary tools:

**Discovery tool:** Performs HTTP GET requests to service URLs, extracts breadcrumb metadata using regex pattern matching, and returns either MCP redirect information or structured API specifications.

**API executor:** Enables direct HTTP requests to discovered endpoints with automatic parameter serialization, supporting GET, POST, PUT, and DELETE methods with configurable timeouts (30 s default).

**Endpoint lister:** Provides quick endpoint enumeration for multi-step workflows.

#### Breadcrumb schema specification

The breadcrumb metadata schema includes service identification (name and version), MCP availability status with optional redirect URLs, and fallback API specifications. Each API endpoint definition specifies the HTTP method, semantic concept tag, parameter schemas with type definition, and example usage URLs.


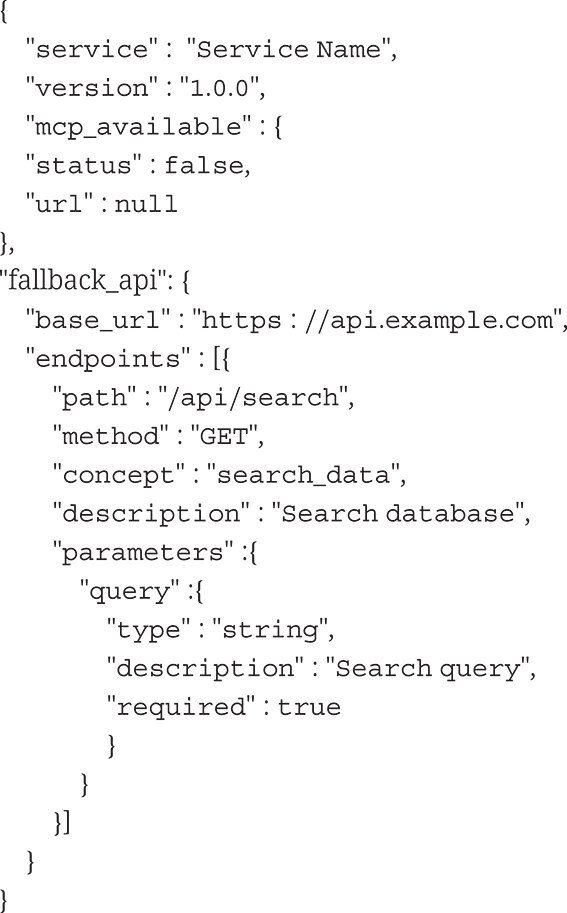


### Validation and error handling

Zod schema validation ensures breadcrumb metadata conforms to specifications before processing. The system implements graceful degradation: if MCP is available, it redirects; if breadcrumbs exist, it exposes API endpoints; otherwise, it indicates manual exploration is required. Network requests include 10-s timeouts, CORS handling, and automatic retry logic for transient failures.

### Integration testing

Functionality was validated using a mock HTTP server (Node.js http module) serving test pages with embedded breadcrumbs. The test suite includes services with MCP availability, breadcrumb-only configurations, and malformed metadata to verify error handling.

## Results

In this manuscript, we introduce **MCPmed** as an open-source hub to share, build, and use MCPs in the biomedical research area. For this, we provide an easy-to-use Cookie cutter template to set up MCP PyPI packages as well as MCP implementations for highly used databases such as GEO, the UCSC Cell Browser [14], STRING DB [29], and PLSDB [30]. In this context, we want to highlight two lightweight applications: first, demonstrating the ease of transitioning existing web services with APIs to MCP services, and second, introducing a practical approach for guiding search engines toward more LLM-native web services.

### GEOmcp

To demonstrate MCP’s practical utility, we implemented a prototype GEOmcp layering over the existing GEO API. This MCP-enhanced service simplifies data discovery and retrieval for autonomous, LLM-driven research. Using straightforward JSON queries, LLM agents autonomously refine search terms contextually, greatly improving search efficiency and success rates compared with manual or traditional keyword-based searches. The implementation includes explicit searches for GEO profiles, GEO datasets, and GEO series. The information required for LLM support is relatively straightforward as shown in the following code snippet describing the functionality for the GEO series search routine:


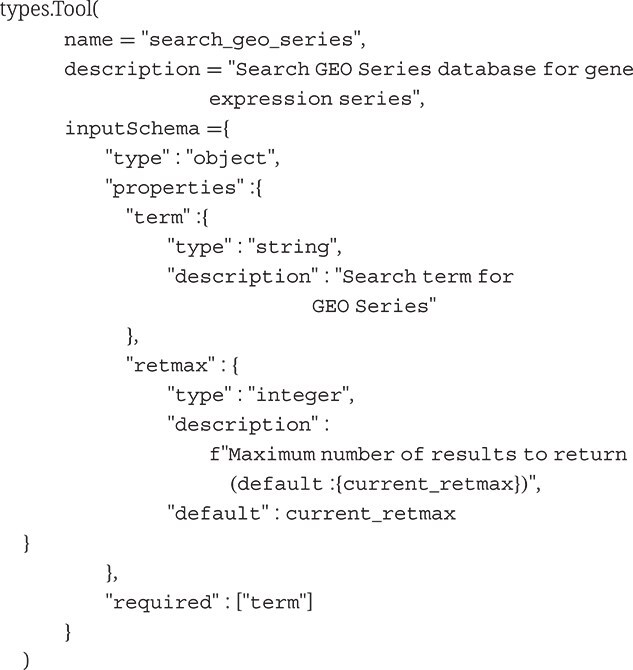


Finally, this example illustrates the autonomous discovery process by LLM agents:

[USER] Hey I am looking for a reference dataset

$\qquad \quad \mathtt{for} \ \mathtt{mice} \ \mathtt{with} \ \mathtt{microglia} \ \mathtt{depletion}$

[CLAUDE] I’ll help you find reference datasets for

$\qquad \quad \mathtt{mice} \ \mathtt{with} \ \mathtt{microglia} \ \mathtt{depletion.}$

$\qquad \quad \mathtt{Let} \ \mathtt{me} \ \mathtt{search} \ \mathtt{the} \ \mathtt{GEO} \ \mathtt{database} \ \mathtt{for}$

$\qquad \quad \mathtt{relevant} \ \mathtt{studies.}$

[CLAUDE] search_geo_queries

$\qquad \quad \mathtt{Request}\{ \mathtt{term:} \ \mathtt{microglia} \ \mathtt{depletion}$

$\qquad \quad \mathtt{mouse,} \ \mathtt{retmax:} \ \mathtt{5} \}$

$\qquad \quad{\texttt{Response}}\{...\mathtt{Empty} \ \mathtt{id} \ \mathtt{list...}\}$

[CLAUDE] Let me try a broader search with

$\qquad \quad \mathtt{different} \ \mathtt{terms:}$

[CLAUDE] search_geo_queries

$\qquad \quad \mathtt{Request}\{ \mathtt{term:} \ \mathtt{microglia} \ \mathtt{PLX3397} \ \mathtt{mouse,}$

$\qquad \quad \mathtt{retmax:} \ \mathtt{5} \}$

$\qquad \quad \mathtt{Response}\{...\mathtt{Empty} \ \mathtt{id} \ \mathtt{list...}\}$

[CLAUDE] Let me try searching for CSF1R inhibition,

$\qquad \quad \mathtt{which} \ \mathtt{is} \ \mathtt{a} \ \mathtt{common} \ \mathtt{method} \ \mathtt{for} \ \mathtt{microglia}$

$\qquad \quad \mathtt{depletion:}$

[CLAUDE] search_geo_queries

$\qquad \quad \mathtt{Request}\{ \mathtt{term:} \ \mathtt{CSF1R} \ \mathtt{inhibitor} \ \mathtt{mouse} \ \mathtt{brain,}$

$\qquad \quad \mathtt{retmax:} \ \mathtt{5} \}$

$\qquad \quad \mathtt{Response}\{...\mathtt{Empty} \ \mathtt{id} \ \mathtt{list...}\}$

[CLAUDE] Let me try a broader microglia-related

$\qquad \quad \mathtt{search:}$

[CLAUDE] search_geo_queries

$\qquad \quad \mathtt{Request}\{ \mathtt{term:} \ \mathtt{microglia} \ \mathtt{mouse} \ \mathtt{brain,}$

$\qquad \quad \mathtt{retmax:} \ \mathtt{10} \}$

$\qquad \quad \mathtt{Response}\{...\mathtt{Empty} \ \mathtt{id} \ \mathtt{list...} \}$

[CLAUDE] search_geo_queries

$\qquad \quad \mathtt{Request}\{ \mathtt{term:} \ \mathtt{microglia} \ \mathtt{depletion,}$

$\qquad \quad \mathtt{retmax:} \ \mathtt{10} \}$

$\qquad \quad{\texttt{Response}}\{ \mathtt{uids:} \ \mathtt{[200299005,} \ \mathtt{200279739,}...] \}$

[CLAUDE] Great! I found several reference datasets

$\qquad \quad \mathtt{for} \ \mathtt{microglia} \ \mathtt{depletion} \ \mathtt{in} \ \mathtt{mice.}$

$\qquad \quad \mathtt{Key} \ \mathtt{datasets:}$

$\qquad \quad \mathtt{GSE271535}$

$\qquad \quad \mathtt{microglia} \ \mathtt{innate} \ \mathtt{immune} \ \mathtt{memory} \ \mathtt{after}$

$\qquad \quad \mathtt{CSF1R} \ \mathtt{depletion} \ \mathtt{(RNA-seq)}$

$\qquad \quad \mathtt{GSE270935}$

$\qquad \quad \mathtt{companion} \ \mathtt{ATAC-seq} \ \mathtt{dataset}$

$\qquad \quad \mathtt{GSE292830}$

$\qquad \quad \mathtt{PLX3397-mediated} \ \mathtt{depletion} \ \mathtt{with}$

$\qquad \quad \mathtt{monocyte} \ \mathtt{replacement}$

$\qquad \quad \mathtt{GSE288018} / \mathtt{GSE288504}$

$\qquad \quad \mathtt{Krabbe} \ \mathtt{disease} \ \mathtt{model} \ \mathtt{with}$

$\qquad \quad \mathtt{microglia} \ \mathtt{replacement}$

$\qquad \quad \mathtt{GSE299005}$

$\qquad \quad \mathtt{post-TBI} \ \mathtt{microglia} \ \mathtt{depletion}$

$\qquad \quad \mathtt{(snRNA-seq)}$

[CLAUDE] For comprehensive coverage,

$\qquad \quad \mathtt{start} \ \mathtt{with} \ \mathtt{GSE271535} \ \mathtt{(RNA-seq)}$

$\qquad \quad \mathtt{and} \ \mathtt{GSE270935} \ \mathtt{(ATAC-seq).}$

This implementation demonstrates MCP’s potential in enabling autonomous contextual refinement, significantly streamlining dataset discovery processes. All code is freely available on PyPI and GitHub (see Section “Code availability”).

### Breadcrumbs

We introduce breadcrumbs, a lightweight HTML-embedded JSON snippet designed for services lacking native MCP or APIs. Breadcrumbs help autonomous agents identify and transition smoothly to MCP-ready alternatives or fallback mechanisms:


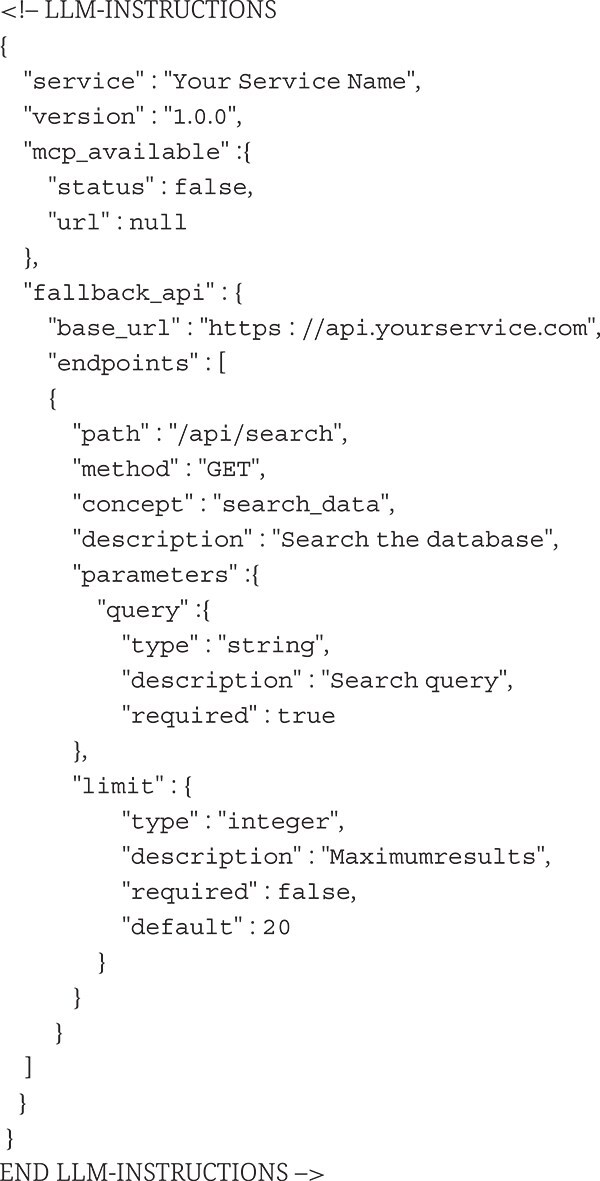


#### Breadcrumbs validation testing

To evaluate the breadcrumbs discovery system’s functionality, we conducted four systematic tests demonstrating its core capabilities (detailed protocols and LLM interaction logs provided in Supplementary Material 2):

**Test 1: No breadcrumbs detection**: The system correctly identified services lacking embedded metadata, returning a “manual exploration required” message without attempting to parse non-existent structures, thus preventing false positives.

**Test 2: Valid breadcrumbs parsing**: Services with properly formatted breadcrumb metadata were successfully parsed, with all endpoint definitions and parameter schemas correctly extracted and formatted for LLM consumption. The system accurately reconstructed API documentation from embedded JSON.

**Test 3: Malformed breadcrumbs handling**: When presented with malformed JSON within breadcrumb comments, the system provided graceful degradation with informative error messages rather than failing silently or producing corrupted outputs. Zod schema validation caught structural errors before they could propagate to LLM interactions.

**Test 4: MCP redirect recognition**: When breadcrumbs indicated native MCP availability (mcp_available.status: true), the system correctly prioritized the native MCP endpoint and provided appropriate recommendations to use the full integration rather than fallback mechanisms.

These tests demonstrate that breadcrumbs can function as a practical discovery mechanism for services in transition to full MCP support. The successful parsing across varied scenarios validates the core technical approach, while revealing important practical constraints.

Breadcrumbs offer a straightforward intermediate solution for rapid MCP adoption, minimizing immediate integration barriers while the community transitions toward native MCP implementations or dedicated metadata endpoints (e.g. /.well-known/llm-manifest.json).

### Cross-service integration: GEOmcp + STRING DB

To demonstrate MCP’s potential for seamless service integration, we conducted a structured workflow combining GEO dataset discovery with STRING protein–protein interaction analysis. The workflow was initiated with a complex, multi-phase research query requiring coordinated use of multiple bioinformatics services (Supplementary Material 1).

#### Experimental design

We tested MCP-enabled service integration through a four-phase research workflow: (i) dataset discovery using GEO, (ii) data analysis setup with gene extraction, (iii) network analysis with STRING, and (iv) biological synthesis integrating findings across services. This design mimics typical bioinformatics research patterns where investigators iteratively query multiple databases to build comprehensive bioinformatics understanding.

#### Phase 1: Autonomous dataset discovery

Given the task to identify “small aging-related mouse brain datasets (preferably with <20 samples),” the GEOmcp layer executed targeted searches. Initial queries for “aging mouse brain small dataset” and “mouse brain aging hippocampus genes” yielded GSE278158, a 6-sample RNA-seq dataset investigating steroid receptor coactivator-1 (SRC-1/NCOA1) in aging-associated cognitive decline. The autonomous selection criteria correctly prioritized: (i) small sample size (six samples), (ii) brain-specific tissue (hippocampus), (iii) explicit gene mentions (NCOA1, S100A6, and S100A11), and (iv) clear pathway annotations (neural plasticity and cognitive decline). This demonstrates MCP’s ability to interpret complex, multi-constraint queries without explicit parameter specification.

#### Phase 2: Automated data extraction and gene compilation

Following dataset selection, the workflow successfully downloaded GSE278158 metadata files (family.soft.gz, metadata.xml) totaling 0.01 MB. From the study description, the system extracted three primary genes (NCOA1, S100A6, and S100A11) and augmented this list with 12 biologically relevant genes based on pathway context: neuroplasticity markers (BDNF, ARC, SYP, and DLG4), immediate early genes (FOS, and EGR1), transcription factors (CREB1), stress response elements (NR3C1), glial markers (GFAP and AIF1), and inflammatory mediators (TNF). This context-aware gene expression, from 3 explicitly mentioned to 15 biologically relevant genes, showcases MCP’s capability to leverage domain knowledge for comprehensive analysis.

#### Phase 3: Network construction and analysis

The 15-gene list was seamlessly transferred to STRING MCP, which achieved 100% mapping success to mouse protein identifiers. Network analysis revealed exceptional biological coherence:

- **Network statistics**: 49 protein–protein interactions observed versus 6 expected by chance (PPI enrichment $P$-value $<10^{-16}$)
- **Hub gene identification**: FOS emerged as the primary hub (12 connections), followed by CREB1 (11), and BDNF (10)
- **Functional enrichment**: Top pathways included regulation of synaptic plasticity (GO:0048167, $P=6.73e-13$), learning or memory (GO:0007611, $P=1.63e-06$), and cAMP signaling (KEGG:mmu04024, $P=2.41e-05$)
- **Network modularity**: Four distinct functional modules were identified, Memory/Plasticity (FOS-CREB1-BDNF-ARC-EGR1), Inflammatory (TNF-AIF1-GFAP), Calcium signaling (S100A6-S100A11), and Nuclear receptor (NCOA1-NR3C1, interaction score 0.951)

#### Phase 4: Integrated biological insights

The coordinated analysis across GEO and STRING revealed NCOA1 as a master regulator positioned upstream of both calcium dysregulation (via S100A6/S100A11) and neuroinflammation (via TNF pathway). The convergence of multiple signaling cascades on CREB1 suggests it functions as a critical integration point for aging-related cognitive decline. The strong connectivity between inflammatory mediator TNF and memory-associated genes (FOS, CREB1, and BDNF) provides mechanistic insight into neuroinflammation’s direct impact on memory circuits.

#### Workflow performance metrics

The complete four-phase workflow executed in $\sim $45 s, involving nine distinct MCP tool calls across two services:

- GEO searches: two queries with automatic result filtering
- Data download: one successful retrieval operation (and 1 status call)
- STRING operations: five calls including identifier mapping, network construction, enrichment analysis, and visualization
- Total data points analyzed: 15 genes, 49 interactions, and 278 GO terms
- Success rate: 100% for all critical operations (gene mapping, network generation, and enrichment calculation)

#### Validation of model context protocol capabilities

This structured workflow demonstrates five key advantages of MCP-enabled service integration:

**1. Context preservation**: Gene selections from GEO were enriched with biological context before STRING analysis, maintaining research focus throughout the workflow.

**2. Automatic format negotiation**: Gene symbols from GEO were seamlessly converted to STRING identifiers without manual intervention or data loss.

**3. Error recovery**: When initial GEO searches returned empty results, the system automatically reformulated queries rather than failing, demonstrating robust error handling.

**4. Semantic understanding**: The system correctly interpreted high-level requirements (“small dataset,” “aging-related”) into appropriate technical parameters.

**5. Cross-service synthesis**: The final integration combined statistical evidence (network *P*-values) with biological annotation (pathway enrichment) to generate testable hypotheses.

The successful execution of this complex, multi-service workflow, from natural language research question to actionable biological insights, validates MCP as a practical solution for integrating heterogeneous bioinformatics resources. The ability to maintain scientific context while traversing multiple databases represents a significant advance over current practices requiring manual data transfer and format conversion between services.

## Conclusion

We demonstrate that adopting MCP enhances bioinformatics web services, making them inherently suitable for LLM-driven automated research. GEOmcp exemplifies immediate benefits, enabling autonomous refinement and precise data retrieval, improving discoverability and contextual precision. The introduced “breadcrumbs” approach serves as a practical transition tool, ensuring legacy web servers [13] can become MCP-ready with minimal effort. Adopting MCP now positions bioinformatics resources for more scalable, reproducible, and efficient research workflows, supporting the evolution toward enhanced biomedical research automation. Implementing this approach may improve machine readability and facilitate better integration into automated workflows as suggested by recent research [2]. Additionally, our group plans to upgrade several bioinformatics databases previously introduced by our chair to become fully MCP-compatible, serving as practical examples and accelerating the broader transition toward fully automated, agent-driven research ecosystems. Finally, the immediate creation of a rigorously curated MCP app store is essential, providing secure, prevetted MCP packages for existing web-services and forming a unified bulwark against misuse and malware across the bioinformatics ecosystem. We already introduced MCPmed in this paper to address this need. As a next intermediate step, we propose automated functionality for data integration templating, paving the way toward a rigorously curated MCP app store offering secure, prevetted packages for existing web services and strengthening protection against misuse and malware across the bioinformatics ecosystem.

### Semantic alignment and ontology integration

While MCP provides the standard protocol for transport and tool discovery, semantic interoperability requires a rigorous controlled vocabulary. Recognizing that general schemas may be insufficient for specialized bioinformatics concepts, MCPmed adopts a hierarchical semantic strategy. At the protocol level, each MCP tool exposes a concept field that is intended not to be a free-text label, but a pointer to one or more stable identifiers from established vocabularies. For generic web entities, we align with *Schema.org* [31] (e.g. “SearchAction” and “Dataset”) and extend this with Bioschemas [6] and the EDAM ontology [7] for domain-specific precision. In our current prototypes, we already implement this pattern but only partially populate it: we use simple string tags such as “search_data” or “download_file” to demonstrate functionality and explicitly treat these as placeholders for the corresponding Schema.org/Bioschemas/EDAM identifiers. A fully mature MCP deployment would instead annotate a search endpoint directly with, e.g. schema:SearchAction and an appropriate EDAM operation term (e.g. an expression-data retrieval operation), ensuring that autonomous agents can interpret both the computational action and its biological context in a consistent way across services and institutions. This separation of concerns, MCP providing the “pipe” and Bioschemas/EDAM providing the “payload,” allows communities to evolve semantic standards without changing the underlying protocol.

## Limitations

### Breadcrumbs discovery constraints

#### Technical fragility

The breadcrumbs approach relies on regex-based extraction of HTML comments, making it vulnerable to certain structural changes in web pages. Our validation testing (Supplementary Material 2) demonstrated successful parsing across four scenarios, including graceful handling of malformed metadata and resilience to cosmetic UI changes in controlled testing. However, the HTML comment-based approach faces challenges with dynamic content generation and complex web architectures. Services that dynamically generate page content, use JavaScript frameworks for client-side rendering, or employ A/B testing may render breadcrumbs inconsistently or omit them entirely during routine updates. Additionally, certain HTML entities, special characters, or nested comment structures can corrupt the JSON payload during parsing, though validation schemas catch many such errors. While we validate extracted metadata using Zod schemas, the extraction phase itself lacks robust error recovery for edge cases. External metadata manifests served via dedicated endpoints (e.g. /.well-known/llm-manifest.json) would provide more reliable discovery but require server-side implementation changes.

#### Semantic ambiguity

The current breadcrumb implementation utilizes simplified concept tags (e.g. “search_data,” “download_file”) to demonstrate functionality. However, these lack a formal controlled vocabulary, which creates potential for ambiguity when integrating services from different providers. As noted in the semantic alignment strategy, this limitation is transient; future iterations of the protocol must enforce strict mapping to Bioschemas profiles and EDAM ontology terms. Without this standardization, autonomous agents may struggle to distinguish between nuances (e.g. “download raw data” vs. “download normalized counts”) if services rely solely on non-standardized string literals.

#### Scalability and maintenance overhead

Embedding identical metadata across multiple pages of a service creates maintenance challenges. Updates to API specifications require coordinating changes across all pages containing breadcrumbs. For large services with hundreds of pages, this redundancy is inefficient and error-prone. A centralized manifest approach would be more maintainable, though it sacrifices the discoverability advantage of embedding metadata directly in user-facing pages.

#### Authentication and state management

The current breadcrumbs implementation does not address authentication flows, session management, or rate limiting policies. Services requiring OAuth, API keys, or cookie-based sessions cannot be fully described through static metadata. While breadcrumbs can document that authentication is required, they cannot facilitate the authentication process itself. This limitation restricts breadcrumbs to public APIs or requires out-of-band credential configuration.

#### Security and trust implications

The ease of adding breadcrumbs introduces novel risks for LLM-mediated research. Maliciously crafted metadata could prime LLMs to preferentially cite certain sources, artificially inflate metrics, or introduce subtle biases in automated analysis. Unlike traditional web services where humans critically evaluate information, LLMs may uncritically incorporate breadcrumb metadata into reasoning chains. This underscores the need for a curated registry (as proposed in MCPmed) where breadcrumb implementations undergo community review before distribution. The lack of cryptographic signatures or publisher verification in the current specification further compounds these trust issues.

### Technical and infrastructure limitations

Technical constraints such as LLM context window limitations, network latency, and evolving proficiency in autonomous tool use still impose practical limitations, although these are expected to decrease over time. Web services without existing API remain hard to bridge to LLMs as, e.g. the UCSC Cell Browser MCP is limited in function.

### Provenance and explainability

While MCP provides a standardized interface for tool invocation, the current GEOmcp implementation does not include comprehensive provenance tracking or explainability mechanisms. In principle, MCP’s structured metadata could serve as a foundation for machine-interpretable audit trails by logging:

- Tool invocation timestamps and parameters
- Data source versions and access patterns
- Intermediate results and transformation steps
- Error states and recovery actions

However, implementing robust provenance requires additional infrastructure beyond the protocol itself, including persistent logging systems, standardized metadata schemas for versioning, and mechanisms for linking related tool calls across multi-step workflows. Our current implementation prioritizes functional integration over comprehensive auditability, leaving full provenance tracking as important future work. This limitation is particularly relevant for research contexts requiring complete reproducibility documentation or regulatory compliance scenarios.

### Model context protocol implementation and model compatibility

While MCP is designed as a model-agnostic protocol, our current implementation and validation focus on Claude (Anthropic) due to practical constraints in the early-stage MCP ecosystem. During development, we explored integration with alternative LLM platforms, including ChatGPT (OpenAI). However, functional implementation required substantial workarounds and custom adapter layers inconsistent with the seamless interoperability MCP envisions. Our implementation leverages Claude Desktop’s native MCP support that currently represents the most mature implementation of the MCP standard available. As native support expands across additional LLM providers, multi-model validation will become increasingly feasible. We emphasize that this limitation reflects the current state of MCP adoption rather than fundamental protocol constraints; our GEOmcp, STRING MCP, and other MCPmed implementations follow MCP specifications strictly and should function with any MCP-compliant client once native support becomes available.

### Configuration complexity and security risks

While there is currently no universally ready-to-use MCP solution requiring zero configuration, recent frameworks such as FastMCP have significantly lowered the barrier to creating MCP servers. Building basic MCP implementations has become relatively straightforward when leveraging these frameworks, often requiring only minimal Python code to expose existing APIs through the MCP protocol. Our GEOmcp implementation demonstrates this accessibility, with the core MCP layer requiring fewer than 500 lines of code to wrap existing NCBI E-utilities endpoints.

However, deployment and integration still present practical challenges. End-user configuration remains a bottleneck, particularly for researchers without systems administration experience who must manually edit configuration files, manage authentication credentials, and troubleshoot PATH issues. The lack of a centralized, one-click installation mechanism comparable with browser extensions or app stores creates friction in adoption.

More critically, the ease of creating MCP servers introduces novel security and integrity risks. Manual curation of web services and generation of breadcrumbs presents completely new vectors for scientific misconduct or LLM exploits: When data insights are generated by LLMs and used unchecked by scientists, maliciously crafted website metadata could prime LLMs to favor certain research outcomes or artificially inflate citations to unrelated work. Unlike traditional web services where human users can critically evaluate presented information, LLM agents may uncritically incorporate biased metadata into their reasoning chains.

These risks underscore the urgent need for a rigorously curated MCP registry or app store, where implementations undergo security review and functional validation before distribution. The MCPmed initiative aims to address this gap by providing vetted, community-reviewed MCP implementations for bioinformatics services. This limitation thus presents a unique opportunity: proactive early adoption and centralized curation of MCP technologies can establish security standards and quality benchmarks as the ecosystem matures, positioning the bioinformatics community advantageously as universal and streamlined solutions inevitably emerge.

**Key Points**

- **Bioinformatics services need machine-actionable interfaces**: Traditional web servers designed for human users create barriers for autonomous research agents and large language models (LLMs), requiring a shift from human-centric to machine-readable platforms.
- **Model context protocol (MCP) bridges the automation gap**: MCP provides a standardized semantic layer over existing APIs that explicitly associates endpoints with scientific concepts and metadata, enabling LLMs to autonomously discover, invoke, and verify bioinformatics services.
- **MCPmed demonstrates practical implementation**: The authors present working MCP implementations for widely used databases (GEO, STRING, UCSC Cell Browser, and PLSDB) and introduce *breadcrumbs* as a lightweight transition solution for services not yet fully MCP-enabled.
- **Enhanced research capabilities through automation**: MCP adoption enables autonomous contextual refinement of searches, significantly improving data discovery efficiency compared with manual keyword-based approaches, as demonstrated by the GEOmcp prototype.
- **Community-driven transition toward automated research**: The proposed MCPmed platform serves as an open-source hub for sharing MCP implementations, providing templates and tools to accelerate the bioinformatics community’s transition toward fully automated, agent-driven research workflows.

## Supplementary Material

supplementary-material_bbag076

## Data Availability

No new biological data were generated in this study. All evaluation logs and outputs generated for the examples and validation tests are provided in the Supplementary Materials and are additionally available in the MCPmed GitHub repositories at the pinned commits listed in Section “Code availability.”

## Code availability

All source code, templates, and future MCP releases can be found at https://github.com/MCPmed. Enrollment for contribution will be available at http://www.mcpmed.org/. For reproducibility while maintaining a single distribution platform, the exact versions used in this manuscript are pinned to immutable Git commits on GitHub:

(i) *breadcrumbsMCP*: https://github.com/MCPmed/breadcrumbsMCP/

commit 8c3a7ca48d731520ee3be3bfad2821c7160e0912

(ii) *breadcrumbsTest*: https://github.com/MCPmed/breadcrumbsTest/

commit 7cdcf834ac70a51e073e88d6b0c06a2d9527de24
